# Gender-specific association of early age-related macular degeneration with systemic and genetic factors in a Japanese population

**DOI:** 10.1038/s41598-017-18487-4

**Published:** 2018-01-15

**Authors:** Mariko Sasaki, Sei Harada, Yumiko Kawasaki, Miki Watanabe, Hidemi Ito, Hideo Tanaka, Ayano Takeuchi, Kazuo Tsubota, Toru Takebayashi, Yuji Nishiwaki, Ryo Kawasaki

**Affiliations:** 1 Keio University, Department of Ophthalmology, 160-0016 Tokyo, Japan; 2 Tachikawa Hospital, 190-8531 Tokyo, Japan; 3 National Institute of Sensory Organs, National Tokyo Medical Center, 152-8902 Tokyo, Japan; 4 Department of Preventive Medicine and Public Health, Keio University, 160-8582 Tokyo, Japan; 5 Yamagata University Graduate School of Medical Science, Department of Public Health, 990-2331 Yamagata, Japan; 6 Nagoya City University Graduate School of Medical Sciences, Department of Public Health, 467-8601 Aichi, Japan; 7 Aichi Cancer Research Institute, Division of Molecular & Clinical Epidemiology, 464-8681 Aichi, Japan; 8 Kishiwada Public Health Center, 596-0076 Osaka, Japan; 9 Toho University, Department of Environmental and Occupational Health, 143-8540 Tokyo, Japan

## Abstract

The Tsuruoka Metabolomics Cohort Study included subjects aged 35–74 years from participants in annual health check-up programs in Tsuruoka, Japan. The gender-specific associations of early age-related macular degeneration (AMD) with systemic and genetic factors was assessed cross-sectionally. Of these, 3,988 subjects had fundus photographs of sufficient quality, and early AMD was present in 12.3% and 10.3% of men and women, respectively. In men, higher levels of high-density lipoprotein cholesterol and lower levels of triglycerides were associated with increased odds of having early AMD after adjusting for potential risk factors (for each 1 mmol/L increase, odds ratio [OR]: 1.61 and 0.78, 95% confidence interval [CI]: 1.17–2.23 and 0.64–0.96, respectively). In women, higher levels of total cholesterol and low-density lipoprotein cholesterol were associated with increased risk of having early AMD (OR: 1.21 and 1.26, 95% CI: 1.01–1.44 and 1.03–1.53, respectively). Sub-analysis demonstrated that women with ARMS2 A69S polymorphisms had a stronger risk for early AMD (OR: 3.25, 95% CI: 2.10–5.04) than men (OR: 1.65, 95% CI: 1.02–2.69). Differential associations of early AMD with both systemic and genetic factors by sex were demonstrated in a Japanese cohort, which suggests that disease process of early AMD could be different by sex.

## Introduction

Age-related macular degeneration (AMD) is a leading cause of visual loss in elderly people worldwide^1^, including in Japanese and other Asians^2^. Currently, Asians comprise 60% of the world’s population, and will eventually contribute the highest global prevalence of AMD by 2040^3^. Therefore, AMD is becoming an increasingly important healthcare problem in Asia.

Racial and ethnic differences in AMD have been recognized between Western populations and Asians. For example, polypoidal choroidal vasculopathy (PCV) is more common in Asians^4^, and wet AMD is more frequent in Japanese than in White population, whereas dry AMD are more frequent in White than in Japanese^5,6^. In addition, neovascular AMD is more common in women compared with men in Western populations^7^, while conversely, Asian women have much lower prevalence of neovascular AMD, approximately 1/3, compared with Asian men^2^.

Some studies have presented that differential risk associations exist for AMD between men and women. Among these risk factors are waist circumference^8^, body mass index (BMI), systolic blood pressure (SBP), physical exercise^9^, and coronary artery disease^10^. These studies suggest that AMD disease development might follow different processes in men and women. Further support arises from observations of an association between younger age at menarche and decreased risk for AMD^11^ and a protective effect of hormone therapies against the development of AMD in women^11^. Risk factors for AMD have been extensively studied in Western populations, whereas fewer studies have been published on risk factors for AMD in Asians^2^. Moreover, little is known regarding gender-specific risk factors for AMD, especially in Asians.

We therefore aimed to examine the cross-sectional associations of early AMD with systemic and genetic factors stratified by sex in a Japanese cohort, the Tsuruoka Metabolomics Cohort Study, with 4,010 participants.

## Results

**Figure 1:**
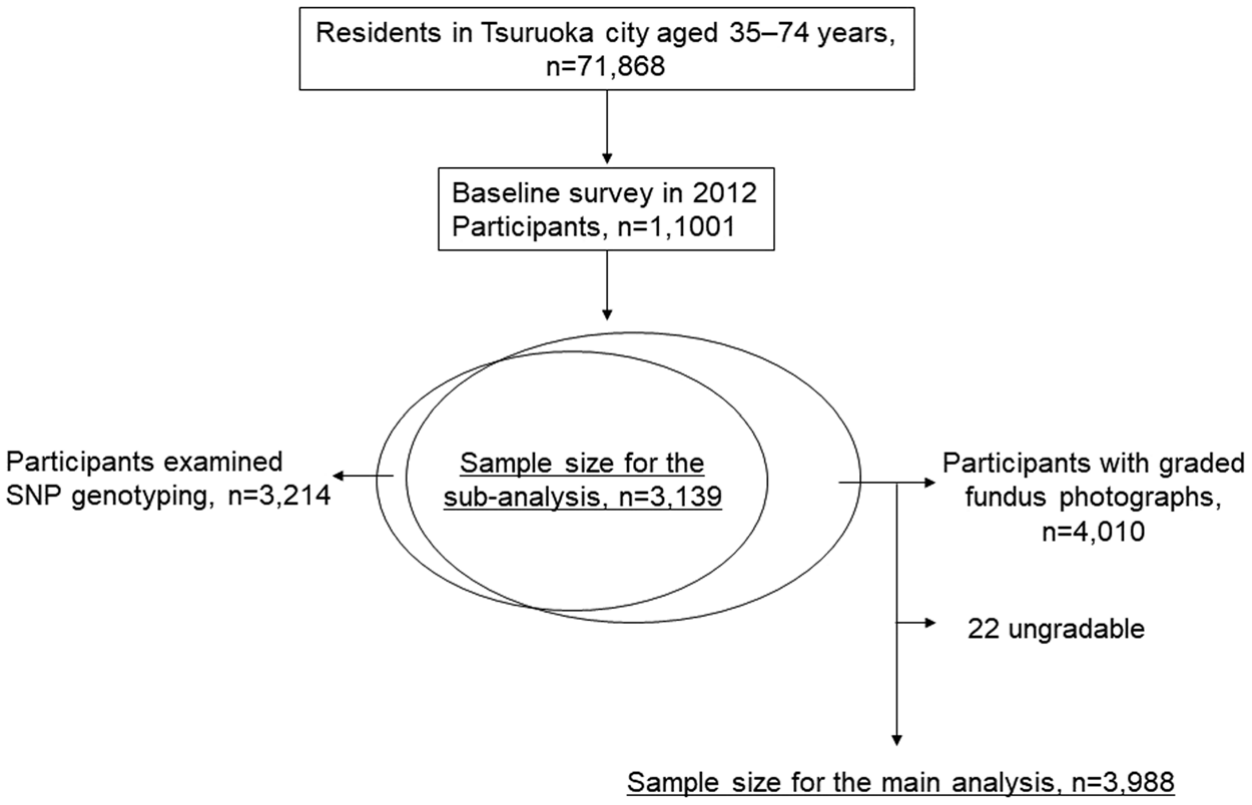
Flowchart describing subject selection from participants of the Tsuruoka Metabolomics Cohort Study.

Among the 4,010 potential subjects, 22 were excluded owing to missing fundus images or suboptimal fundus image quality (i.e., poor focus, lashes, or uneven illumination) and the remaining 3,988 participants (99.5% of the 4,010 participants) (mean age: 62.4 ± 7.6 years) were included as subjects in this analysis (Fig. 1). There were 1,815 (45.5%) male and 2,173 (54.5%) female subjects. There were no differences in the factors between those included in the analyses and those excluded (data not shown). All three variants was in Hardy-Weinberg equilibrium (HWE) in the control group, early AMD and all subjects by sex (P > 0.05).

### Age-specific prevalence of early and late AMD stratified by sex

Table 1 shows the prevalence of specific AMD lesions by age and sex. The prevalence of early AMD was 11.8% among participants aged 50 years and over. Early AMD was more frequent in men than in women (P = 0.030). Overall prevalence of large drusen and pigmentary abnormalities were 11.1% and 0.4%, respectively. Men were more likely to have large drusen and pigmentary abnormalities than women (P = 0.045 and 0.018, respectively). The prevalence of late AMD was 0.054% among those aged 50 years and over. The sub-types of late AMD were all neovascular AMD, and were prevalent only in men (n = 2).

**Table 1:** Age-specific prevalence of early and late AMD according to sex.

	Large drusen	Pigmentary abnormalities	Early AMD	Late AMD	Any AMD
**Men, no. (%)**					
−49, n = 160	10 (6.3)	1 (0.6)	11 (6.9)	0 (0)	11 (6.9)
50–59, n = 385	32 (8.3)	0 (0)	32 (8.3)	0 (0)	32 (8.3)
60–69, n = 955	119 (12.5)	9 (0.9)	121 (12.7)	1 (0.1)	122 (12.8)
70–74, n = 315	60 (19.0)	2 (0.6)	60 (19.0)	1 (0.3)	61 (19.4)
Total, n = 1815	221 (12.2)	12 (0.7)	224 (12.3)	2 (0.1)	226 (12.5)
**Women, no. (%)**					
−49, n = 150	2 (1.3)	0 (0)	2 (1.3)	0 (0)	2 (1.3)
50–59, n = 409	19 (4.6)	0 (0)	19 (4.6)	0 (0)	19 (4.6)
60–69, n = 1217	130 (10.7)	2 (0.2)	130 (10.7)	0 (0)	130 (10.7)
70–74, n = 397	72 (18.1)	2 (0.5)	72 (18.1)	0 (0)	72 (18.1)
Total, n = 2173	223 (10.3)	4 (0.2)	223 (10.3)	0 (0)	223 (10.3)
**Total, no (%)**					
−49, n = 310	12 (3.9)	1 (0.3)	13 (4.2)	0 (0)	13 (4.2)
50–59, n = 794	51 (6.4)	0 (0)	51 (6.4)	0 (0)	51 (6.4)
60–69, n = 2172	249 (11.5)	11 (0.5)	251 (11.6)	1 (0.05)	252 (11.6)
70–74, n = 712	132 (18.5)	4 (0.6)	132 (18.5)	1 (0.1)	113 (18.7)
Total, n = 3988	444 (11.1)	16 (0.4)	447 (11.2)	2 (0.05)	449 (11.3)

### Characteristics of the study participants with early AMD stratified by sex

Characteristics of participants with early AMD by sex are presented in Table 2. Participants with early AMD were significantly older than those without, both in men and women (P < 0.0001, for all). In men, participants with early AMD had significantly lower levels of triglycerides (TG; P < 0.01) and higher levels of high-density lipoprotein cholesterol (HDLC) compared with those without (P < 0.01). In women, participants with early AMD had higher levels of BMI (P = 0.010), and were likely to have hypertension, dyslipidemia, and use of lipid-lowering medication (P < 0.01, for all). Current/past smokers were less likely to have early AMD (P = 0.022), however, the smokers in women were younger than those in men (P < 0.0001).

**Table 2:** Basic characteristics according to sex.

Variables	Men			Women		
	Early AMD, n = 224	Control, n = 1591	P value	Early AMD, n = 223	Control, n = 1950	P value
	No. (%) or Mean ± SD	No. (%) or Mean ± SD		No. (%) or Mean ± SD	No. (%) or Mean ± SD	
Age, y	64.5 ± 7.1	61.7 ± 8.0	**<0.0001**	66.2 ± 5.3	62.2 ± 7.4	**<0.0001**
BMI, kg/m^2^	23.4 ± 2.8	23.7 ± 3.1	0.31	23.4 ± 3.3	23.0 ± 3.4	**0.010**
Smoking history						
Current/past	181 (80.8)	1290 (81.1)	0.93	12 (5.4)	198 (10.1)	**0.022**
Never	43 (19.2)	301 (18.9)		211 (94.6)	1752 (89.9)	
Hypertension	121 (54.0)	854 (53.7)	0.92	127 (57.0)	892 (45.7)	**0.0015**
Dyslipidemia	95 (42.4)	800 (50.3)	**0.027**	143 (64.1)	1055 (54.1)	**0.0044**
Diabetes	22 (9.8)	218 (13.7)	0.11	16 (7.2)	139 (7.1)	0.98
Antihypertension medication	83 (37.1)	515 (32.4)	0.16	78 (35.0)	574 (29.4)	0.087
Lipid-lowering medication	28 (12.5)	223 (14.0)	0.54	77 (34.5)	500 (25.6)	**0.0044**
Total cholesterol, mmol/L	202.2 ± 31.5	205.3 ± 34.7	0.27	217.9 ± 32.6	214.7 ± 32.2	0.14
HDL cholesterol, mmol/L	67.3 ± 16.1	63.4 ± 17.0	**<0.0001**	71.8 ± 16.3	72.2 ± 16.6	0.71
LDL cholesterol, mmol/L	113.0 ± 29.3	116.4 ± 30.9	0.17	127.2 ± 31.1	123.6 ± 29.2	0.077
Triglyceride, mmol/L	110.4 ± 60.6	131.0 ± 97.8	**0.0015**	94.3 ± 41.5	94.9 ± 46.5	0.82

Significant values are in bold.

### Associated factors of early AMD stratified by sex

Table 3 shows associations of early AMD by sex. After adjusting for age, in men, higher levels of HDLC were associated with an increased likelihood of the presence of early AMD (for each 1 mmol/L increase, odds ratio [OR]: 1.60, 95% confidence intervals [CI]: 1.18–2.16), whereas higher levels of TG were associated with a reduced likelihood of the presence of early AMD (for each 1 mmol/L increase, OR: 0.77, 95% CI: 0.63–0.94) (Table 3, Model 1). In women, higher levels of low-density lipoprotein cholesterol (LDLC) were associated with an increased likelihood of the presence of early AMD (for each 1 mmol/L increase, OR: 1.21, 95% CI: 1.01–1.46) (Table 3, Model 1).

**Table 3:** Logistic regression analysis of factors for early AMD according to sex.

Variables	Men						Women					
	Model 1			Model 2			Model 1			Model 2		
	Odds	95% CI	P-value	Odds	95% CI	P-value	Odds	95% CI	P-value	Odds	95% CI	P-value
Age, per 1 year	**1.05**	**1.03–1.08**	**<0.0001**	**1.06**	**1.03–1.08**	**<0.0001**	**1.10**	**1.08–1.13**	**<0.0001**	**1.10**	**1.07–1.13**	**<0.0001**
BMI, per 1 kg/m^2^	0.98	0.93–1.03	0.41	1.00	0.95–1.05	0.93	0.99	0.94–1.04	0.15	1.03	0.981–1.07	0.24
Smoking history,	1.09	0.76–1.56	0.65	1.13	0.78–1.62	0.53	0.85	0.46–1.58	0.61	0.84	0.45–1.56	0.58
Current/past vs Never												
Hypertension	0.88	0.66–1.17	0.37	0.93	0.69–1.25	0.59	1.21	0.90–1.61	0.20	1.15	0.85–1.545	0.37
Dyslipidemia	0.77	0.58–1.27	0.075	0.80	0.60–1.08	0.14	1.31	0.98–1.75	0.071	1.28	0.95–1.71	0.10
Diabetes	0.63	0.39–1.00	0.050	0.66	0.41–1.06	0.080	0.79	0.46–1.36	0.40	0.73	0.42–1.26	0.25
Total cholesterol, per 1 mmol/L	0.95	0.81–1.12	0.57	0.95	0.80–1.12	0.50	1.17	0.98–1.38	0.078	**1.21**	**1.01–1.44**	**0.034**
HDL cholesterol, per 1 mmol/L	**1.60**	**1.18–2.16**	**0.0026**	**1.61**	**1.17–2.23**	**0.0043**	1.01	0.73–1.41	0.94	1.08	0.76–1.53	0.64
LDL cholesterol, per 1 mmol/L	0.91	0.76–1.09	0.28	0.89	0.74–1.07	0.22	**1.21**	**1.01–1.46**	**0.043**	**1.26**	**1.03–1.53**	**0.021**
Triglyceride, per 1 mmol/L	**0.77**	**0.63–0.94**	**0.010**	**0.78**	**0.64–0.96**	**0.019**	0.94	0.71–1.24	0.64	0.90	0.67–1.21	0.44

HDL: high-density lipoprotein, LDL: low-density lipoprotein; Significant values are in bold. Model 1: Adjusted for age. Model 2: For serum lipid: adjusted for age, BMI, smoking history, hypertension, diabetes, and lipid-lowering medication. For the others: adjusted for age, BMI, smoking history, hypertension, dyslipidemia, and diabetes.

Moreover, after adjusting for age, BMI, smoking history, hypertension, diabetes, and lipid-lowering medication, in men, higher levels of HDLC and lower levels of TG remained to be associated with an increased likelihood of the presence of early AMD (Table 3, Model 2). In women, higher levels of LDLC remained to be associated with an increased likelihood of the presence of early AMD. Moreover, each 1 mmol/L increase in total cholesterol (TC) was associated with 1.21 times the increased likelihood of the presence of early AMD (95% CI: 1.01–1.44; Table 3).

Finally, Table 4 shows genetic characteristics by sex. In men, *ARMS2 A69S* TT genotype was found in 16.7% and associated with increased odds of early AMD (OR: 1.65, 95% CI: 1.02–2.69), while *CFH I62V* TT genotype was found in 17.8% of men and not associated with the risk (OR: 1.43, 95% CI: 0.88–2.32). In women, *ARMS2 A69S* TT and *CFH I62V* TT genotypes were more likely to be present than in men in 27.1% and 25.4% respectively, which had higher odds of early AMD than in men (OR: 3.25 and 2.04, 95%CI: 2.10–5.04 and 1.33–3.14, respectively). An association between the *CFH Y402H* variant and AMD has not been detected in this study (data not shown).

**Table 4:** Genetic risk for early AMD according to sex.

Genotype	Prevalence (%)	Men				Prevalence (%)	Women			
		Control, n = 1236	Early AMD, n = 174				Control, n = 1548	Early AMD, n = 181		
		No. (%)	No. (%)	Odds (95% CI)	P-value		No. (%)	No. (%)	Odds (95% CI)	P-value
rs10490924/ARMS2										
GG	10.7	500 (40.5)	60 (34.5)	1 (reference)		7.4	604 (39.0)	48 (26.5)	1 (reference)	
TG	12.6	589 (47.7)	85 (48.8)	1.21 (0.85–1.72)	0.716	10.0	753 (48.6)	84 (46.4)	1.38 (0.95–2.01)	0.0979
TT	16.5	147 (11.9)	29 (16.7)	1.65 (1.02–2.69)	0.069	20.4	191 (12.3)	49 (27.1)	**3.25 (2.10–5.04)**	**<0.0001**
rs800292/CFH I62V										
CC	9.9	456 (36.9)	50 (28.7)	1 (reference)		8.5	559 (36.1)	52 (28.7)	1 (reference)	
CT	13.8	582 (47.1)	93 (53.5)	1.45 (1.01–2.1)	0.2425	9.8	760 (49.1)	83 (45.9)	1.12 (0.77–1.61)	0.126
TT	13.5	198 (16.0)	31 (17.8)	1.43 (0.88–2.32)	0.4299	16.7	229 (14.8)	46 (25.4)	**2.04 (1.33–3.14)**	**0.0005**

Adjusted for age; Significant values are in bold.

## Discussion

We found differential associations of early AMD with systemic and genetic factors among women compared with men. In men, higher levels of HDLC and lower levels of TG were associated with an increased likelihood of the presence of early AMD. In women, higher levels of TC and LDLC were associated with an increased likelihood of the presence of early AMD. Further, having the *CFH I62V* and *ARMS2 A69*S polymorphisms, known polymorphisms for AMD in Asians, might be higher risk for early AMD in women than in men.

Soft drusen is well known to be a main constituent of early AMD and an early sign of AMD. Retinal Pigment Epithelium (RPE) persistently secretes apoB/apoE-containing lipoprotein, which accumulates on Bruch membrane’s surface^12^. These lipoproteins are oxidized or modified in many ways, associated with the formation of soft drusen^13^. The cholesterol-related genes were found to be associated with AMD in genome-wide association studies (GWAS), which suggests that these variants may play important roles in early AMD^14,15^.

AMD can be compared with atherosclerotic cardiovascular disease (CVD) in many ways. For example, by-products of lipoproteins found in atherosclerotic plaque^16^ have also been observed in drusen^12^. HDL transports cholesterol from peripheral tissues to liver, which causes plaque regression and improved endothelial function^17^. Thus, HDL has been known to protect from coronary heart disease^18^. However, in a meta-analysis^19^ and other epidemiological studies^20,21^, high level of HDLC has been reported to result in a significant increase of AMD risk. These findings, and ours as well, are likely to appear inconsistent with known HDL functions. One should consider the possibility that cholesteryl ester transfer protein (CETP) could be involved in this pathological process. CETP is an enzyme that facilitates the exchange of mainly cholesteryl acylester (CE) and TG among lipoproteins such as LDL and HDL^22^. Recently, Burgess S, *et al*.^23^ have indicated that HDLC was a causal risk factor for AMD using Mendelian randomization, and suggests that *CETP* variants could cause AMD through increasing HDLC. Further, *CETP (D442G)* was shown to increase AMD risk by 1.70 times in the genome-wide association studies (GWAS)^24^, which is East Asian-specific and highly present in 6–7% of the Japanese^19,25^. That is also known to impair CETP function and increase dysfunctional HDL^25,26^, and consequently could cause an accumulation of peroxidized lipids on Bruch’s membrane and eventually form drusen^12^. Meanwhile, CETP levels were reported to be higher in women as compared with men^27^. These observations might be related with our gender-specific findings.

Conversely, higher levels of TG were associated with a reduced likelihood of the presence of early AMD in men, which is consistent with some other epidemiological studies^28,29^ and the meta-analysis mentioned above^19^. Since *CETP* variants were reported to increase HDLC and decrease TG, it would be a possibility of reverse causation^23^.

The association between LDLC levels and risk of early AMD is controversial^30,31^; however, the association between LDLC levels and early AMD in the women of our study population is consistent with some previous studies^30^ and known LDLC roles in atherosclerosis. In the retina, there are many genes involved in the biosynthesis and uptake of cholesterol from systemic circulation to preserve cholesterol homeostasis and retinal function^32^, and LDL seems to be the major carrier of cholesterol to the retina^32^. Also, estrogen has antioxidant properties and anti-inflammatory effects that protect against CVD, atherosclerosis, and lipid metabolism, as well as AMD^33^. Among Japanese, LDLC levels are lower in women than in men until their 40’s, and then gradually increase and surpass those in men^34^. Large amounts of cholesterol might overwhelm the cholesterol-regulating system in the retina among the elderly women. Moreover, the increase of CETP levels was suggested to be associated with enhanced peripheral cholesterol transport via low density lipoprotein, etc^35^. It may expand the gender difference through an increase of CETP levels in women.

Regarding genetic factors, our findings as well as previous studies^35,36^ confirmed that the *ARMS2 A69S* polymorphisms are associated with early AMD in Japanese. An association between the *CFH Y402H* variant and AMD, which has been reported in many Western countries^37^, has not been detected in this study as the previous Asian studies^38–40^. It is not surprising since the risk allele frequency in Asians was much lower than in White population^41^, although the meta-analysis proved the association between late AMD and the *CFH Y402H* variant in Asians^41^. Also, we found the association between early AMD and the *CFH I62V* variant in women, however, the direction of the risk allele was inverse to some previous studies of early and late AMD^35,42^. Adams M, *et al*.^43^ reported associations between early AMD and single nucleotide polymorphisms (SNPs) were strongly modified by age, and an inverse association between the high-risk homozygote (CC) for *CFH I62V* and early AMD was observed in younger age, and a positive association was only seen aged >75. In our study, all participants were aged <75, which could partly explain the association we observed. Furthermore, the *CFH I62V* variant^39,40,44^ and risk alleles both in *ARMS2* and *CFH I62V* were more frequently seen in women than in men and higher risk of early AMD for women than for men. These results suggest that known genetic polymorphisms for AMD in Asians could be associated with higher risk of early AMD in women than in men, and different risk factors from women such as *CETP* polymorphism might contribute to the disease process in men.

Strengths of this study included accurate measurements of blood samples with exact fasting state, as well as the use of standardized grading protocols to define AMD by trained graders. The validated questionnaires enabled detailed analysis of the associations between early AMD and serum lipid levels using information on medical histories. We also recognize several limitations with our study. First, the study was a cross-sectional observation, without temporal information of the associations. Second, only one non-mydriatic fundus photograph was taken from a single eye of each participant examined in the study. This could have led to an underestimation of the AMD prevalence per person. Third, because the participants with late AMD were few due to their age (35–74 years old) or potential healthy screenee bias, we could not analyze the association between late AMD and factors. Fourth, participant rate was relatively low especially under 60 years old. The distribution of the age of participants in the current study is not so compatible with that of residents in Tsuruoka city. Fifth, the lipid metabolism-associated genes were not analyzed. Finally, there could be potentially remained confounding associations caused by unmeasured potential confounders such as life styles including nutritional intake^45,46^, therefore future studies to validate these findings are needed.

In conclusion, we analyzed a Japanese cohort with 3988 participants and showed differential associations of early AMD with systemic and genetic factors among women compared with men. These findings suggest that disease process of early AMD could be different by sex. Although prospective longitudinal studies are warranted to confirm this observation, if proven, these findings would contribute to the understanding of the mechanisms of AMD pathology, and reveal interventional options to prevent or slow disease incidence or progression.

## Methods

This study was conducted in accordance with the Ethical Guidelines for Medical and Health Research Involving Human Subjects, Japan, and approved by the Medical Ethics Committees of the School of Medicine, Keio University, Tokyo, Japan (Approval No. 20110264) and the School of Medicine, Toho University, Tokyo, Japan (Approval No. 26028). Written informed consent was obtained from all individual participants included in the study.

### Study population

This study was based on data derived from participants of the Tsuruoka Metabolomics Cohort Study, details of which have been previously described^47^. In brief, between April 2012 and March 2015, 71,868 residents subscribed in the national health insurance in Tsuruoka, Japan, aged 35–74 years were identified and invited to the annual municipal health check-up programs who included fundus photographs. Accordingly, 11,001 subjects were enrolled from the participants (participant rate, 15.3%). In this analysis, a total of 4,010 individuals who participated in the baseline survey between April 2012 and March 2013 were included (Fig. 1).

### Data and sample collection

Each participant underwent a comprehensive assessment including a range of clinical, biochemical, and anthropometric measurements, and lifestyle factors collected from validated questionnaires. All data and samples were obtained during annual health check-ups.

Non-stereoscopic fundus photographs of one eye (generally the right eye) were obtained using a 45° non-mydriatic fundus camera (TRC-NW200 [TOPCON Corp., Tokyo, Japan]) without using pharmacological dilating agents. Images were centered on the optic disc and macula. If the fundus photography of the right eye was not possible because of media opacity or other reasons, photographs of the left eye were taken (n = 126).

BP was measured twice after participants were seated comfortably for at least 5 minutes. The mean of two measures of systolic and diastolic BP was used for analysis. BMI was calculated as weight (kilograms) divided by the square of height (meters).

Blood samples were collected in the morning between 8:30 am and 10:30 am after overnight fasting to avoid variation due to fasting state and circadian rhythm. Blood glucose, hemoglobin A1c (HbA1c, %), and lipids (TC, HDLC, LDLC and TG, mg/dL and mM/L) were measured. Serum lipid levels of the fasting blood samples were measured using an enzyme assay, which had been confirmed to be precise and valid for standardized testing by the Japan Medical Association^48^. The LDLC levels were calculated using the Friedenwald formula where plasma TG concentrations were less than or equal to 4.5 mM/L (400 mg/dL). 28 participants were excluded due to high concentrations of TG.

Information regarding smoking habits and alcohol intake, and history of antihypertension, antidiabetic and lipid-lowering medications was obtained using a standardized self-administered questionnaire. Hypertension was defined as having systolic BP ≥140 mmHg, diastolic BP ≥90 mmHg^49^, or a use of antihypertension medication. Diabetes was defined as having HbA1c (NGSP) ≥6.5%^50^ or a use of antidiabetic medication. Dyslipidemia was defined as having LDLC ≥140 mg/dl, HDLC <40 mg/dl, TG ≥150 mg/dlor^51^, or a use of lipid-lowering medication.

### Grading of Fundus Photographs for Age-Related Macular Degeneration

A non-mydriatic fundus photograph of one eye of each participant was evaluated to determine whether quality was sufficient for grading of AMD lesions. These fundus photographs were evaluated at the reading center of Yamagata University (Principal Investigators: RK YK). Details of the AMD photograph grading followed protocols used for the Blue Mountain Eye Study (BMES), as described elsewhere^52^. In brief, a trained grader (YK) assessed photographs for AMD signs in masked fashion, following the modified Wisconsin Age-Related Maculopathy Grading System^53^ protocol used in the BMES^52^, with a super-vision by a senior researcher (RK) when uncertainties in the grading occurred.

Early AMD was defined as the presence of a large drusen (soft distinct or soft indistinct drusen with a diameter >125 μm and/or RPE abnormality (hyperpigmentation or hypopigmentation) within the grid (a 3000-μm radius centered on the fovea), in the absence of late AMD^6,53^. Late AMD was defined as the presence of exudative AMD or geographic atrophy (GA). Exudative AMD was defined as the presence of subretinal or sub-RPE hemorrhage, RPE detachment, serous detachment of the sensory retina or subretinal fibrous scars^6^. Geographic atrophy was defined as sharply edged, roughly round, or oval areas of RPE loss, with clearly visible choroidal vessels^6^.

### Genotyping

Genomic DNA was extracted from the buffy coat fraction in accordance with standard procedures using a phenol-chloroform extraction method and checked for quality using Qubit (Life Technologies).

Participants were tested for three major AMD-associated SNPs: *ARMS2 A69S* (rs10490924), *CFH I62V* (rs800292), and *CFH Y402H* (rs1061170) using SNP Type Assays (Fluidigm, San Francisco, CA, USA). The quality control of genotyping was assessed statistically using the Hardy-Weinberg test, and P values more than 0.05 were considered that genotype distributions were in HWE. 5% random-samples were retyped by two different examiners, and those were 100% matched.

### Statistical Analysis

The age- and sex-specific prevalence of early AMD and late AMD was calculated, and the subjects were classified as having either early or late AMD if they had one sign of early or late AMD. Baseline characteristics were compiled for overall samples and also for subgroups stratified by sex. Differences in basic characteristics between sexes were assessed using Wilcoxon rank-sum test for continuous variables since they were not normally distributed, and χ2 test or Fisher’s exact test for categorical variables.

Associations of early AMD were assessed using two multivariable logistic regression models. The first model was to determine associations between the presence of early AMD and serum lipid adjusting for age, BMI, smoking history, hypertension, diabetes, and lipid-lowering medication. The second model was to determine associations between the presence of early AMD and the other factors adjusting for age, BMI, smoking history, hypertension, dyslipidemia and diabetes. Associations between the presence of early AMD and genetic factors were assessed using multivariable logistic regression models adjusted for age. The associations were expressed as ORs with CIs. P values less than 0.05 were considered statistically significant. SAS version 9.4 for Windows (SAS Institute Inc., Cary, NC, USA) was used to perform all statistical analyses.

## References

[1] Lim LS, Mitchell P, Seddon JM, Holz FG, Wong TY (2012). Age-related macular degeneration. Lancet.

[2] Kawasaki R (2010). The prevalence of age-related macular degeneration in Asians: a systematic review and meta-analysis. Ophthalmology.

[3] Wong WL (2014). Global prevalence of age-related macular degeneration and disease burden projection for 2020 and 2040: a systematic review and meta-analysis. Lancet Glob Health.

[4] Maruko I, Iida T, Saito M, Nagayama D, Saito K (2007). Clinical characteristics of exudative age-related macular degeneration in Japanese patients. Am J Ophthalmol.

[5] Klein R, Klein BE, Linton KL (1992). Prevalence of age-related maculopathy. The Beaver Dam Eye Study. Ophthalmology.

[6] Oshima Y (2001). Prevalence of age related maculopathy in a representative Japanese population: the Hisayama study. The British journal of ophthalmology.

[7] Rudnicka AR (2012). Age and gender variations in age-related macular degeneration prevalence in populations of European ancestry: a meta-analysis. Ophthalmology.

[8] Munch IC, Linneberg A, Larsen M (2013). Precursors of age-related macular degeneration: associations with physical activity, obesity, and serum lipids in the inter99 eye study. Invest Ophthalmol Vis Sci.

[9] Erke MG (2014). Cardiovascular risk factors associated with age-related macular degeneration: the Tromso Study. Acta Ophthalmol.

[10] Wang SB (2015). Severity of coronary artery disease is independently associated with the frequency of early age-related macular degeneration. The British journal of ophthalmology.

[11] Snow KK, Cote J, Yang W, Davis NJ, Seddon JM (2002). Association between reproductive and hormonal factors and age-related maculopathy in postmenopausal women. Am J Ophthalmol.

[12] Pikuleva IA, Curcio CA (2014). Cholesterol in the retina: the best is yet to come. Prog Retin Eye Res.

[13] Spaide RF, Armstrong D, Browne R (2003). Continuing medical education review: choroidal neovascularization in age-related macular degeneration–what is the cause?. Retina.

[14] Neale BM (2010). Genome-wide association study of advanced age-related macular degeneration identifies a role of the hepatic lipase gene (LIPC). Proc Natl Acad Sci USA.

[15] Fritsche, L. G. *et al*. Seven new loci associated with age-related macular degeneration. *Nat Genet* **45**, 433–439, 439e431–432, 10.1038/ng.2578 (2013). 10.1038/ng.2578 PMC3739472 23455636

[16] Upston JM (2002). Disease stage-dependent accumulation of lipid and protein oxidation products in human atherosclerosis. Am J Pathol.

[17] Rader DJ, Tall AR (2012). The not-so-simple HDL story: Is it time to revise the HDL cholesterol hypothesis?. Nat Med.

[18] Gordon T, Kannel WB, Castelli WP, Dawber TR (1981). Lipoproteins, cardiovascular disease, and death. The Framingham study. Arch Intern Med.

[19] Wang Y (2016). The Association between the Lipids Levels in Blood and Risk of Age-Related Macular Degeneration. nutrients.

[20] van Leeuwen R (2004). Cholesterol and age-related macular degeneration: is there a link?. Am J Ophthalmol.

[21] Delcourt C (2001). Associations of cardiovascular disease and its risk factors with age-related macular degeneration: the POLA study. Ophthalmic Epidemiol.

[22] Ohnishi T, Tan C, Yokoyama S (1994). Selective transfer of cholesteryl ester over triglyceride by human plasma lipid transfer protein between apolipoprotein-activated lipid microemulsions. Biochemistry.

[23] Burgess S, Davey Smith G (2017). Mendelian Randomization Implicates High-Density Lipoprotein Cholesterol-Associated Mechanisms in Etiology of Age-Related Macular Degeneration. Ophthalmology.

[24] Cheng CY (2015). New loci and coding variants confer risk for age-related macular degeneration in East Asians. Nat Commun.

[25] Inazu A (1994). Genetic cholesteryl ester transfer protein deficiency caused by two prevalent mutations as a major determinant of increased levels of high density lipoprotein cholesterol. J Clin Invest.

[26] Takahashi K (1993). A missense mutation in the cholesteryl ester transfer protein gene with possible dominant effects on plasma high density lipoproteins. J Clin Invest.

[27] Marcel YL (1990). Distribution and concentration of cholesteryl ester transfer protein in plasma of normolipemic subjects. J Clin Invest.

[28] Cackett P (2008). Smoking, cardiovascular risk factors, and age-related macular degeneration in Asians: the Singapore Malay Eye Study. Am J Ophthalmol.

[29] Klein R (2003). Early age-related maculopathy in the cardiovascular health study. Ophthalmology.

[30] Colak E (2011). The association of lipoprotein parameters and C-reactive protein in patients with age-related macular degeneration. Ophthalmic Res.

[31] Wang, Y. *et al*. The Association between the Lipids Levels in Blood and Risk of Age-Related Macular Degeneration. *Nutrients* **8**, 10.3390/nu8100663 (2016). 10.3390/nu8100663 PMC5084049 27782072

[32] Tserentsoodol N (2006). Uptake of cholesterol by the retina occurs primarily via a low density lipoprotein receptor-mediated process. Mol Vis.

[33] Barton M (2013). Cholesterol and atherosclerosis: modulation by oestrogen. Curr Opin Lipidol.

[34] Arai H (2005). Serum lipid survey and its recent trend in the general Japanese population in 2000. J Atheroscler Thromb.

[35] Aoki A (2015). Risk Factors for Age-Related Macular Degeneration in an Elderly Japanese Population: The Hatoyama Study. Invest Ophthalmol Vis Sci.

[36] Holliday EG (2013). Insights into the genetic architecture of early stage age-related macular degeneration: a genome-wide association study meta-analysis. PLoS One.

[37] Klein RJ (2005). Complement factor H polymorphism in age-related macular degeneration. Science.

[38] Gotoh N (2006). No association between complement factor H gene polymorphism and exudative age-related macular degeneration in Japanese. Hum Genet.

[39] Mori K (2007). Coding and noncoding variants in the CFH gene and cigarette smoking influence the risk of age-related macular degeneration in a Japanese population. Invest Ophthalmol Vis Sci.

[40] Ng TK (2008). Multiple gene polymorphisms in the complement factor h gene are associated with exudative age-related macular degeneration in chinese. Invest Ophthalmol Vis Sci.

[41] Kondo N, Bessho H, Honda S, Negi A (2011). Complement factor H Y402H variant and risk of age-related macular degeneration in Asians: a systematic review and meta-analysis. Ophthalmology.

[42] Hayashi H (2010). CFH and ARMS2 variations in age-related macular degeneration, polypoidal choroidal vasculopathy, and retinal angiomatous proliferation. Invest Ophthalmol Vis Sci.

[43] Adams MK (2012). Can genetic associations change with age? CFH and age-related macular degeneration. Hum Mol Genet.

[44] Yamashiro K (2011). Association of elastin gene polymorphism to age-related macular degeneration and polypoidal choroidal vasculopathy. Invest Ophthalmol Vis Sci.

[45] SanGiovanni JP (2008). The relationship of dietary omega-3 long-chain polyunsaturated fatty acid intake with incident age-related macular degeneration: AREDS report no. 23. Arch Ophthalmol.

[46] Age-Related Eye Disease Study Research G (2007). The relationship of dietary carotenoid and vitamin A, E, and C intake with age-related macular degeneration in a case-control study: AREDS Report No. 22. Arch Ophthalmol.

[47] Sei Harada TT (2016). Metabolomic profiling reveals novel biomarkers of alcohol intake and alcohol-induced liver injury in community-dwelling men. Environ Health Prev Med.

[48] Kuwabara K (2016). Relationship between Non-High-Density Lipoprotein Cholesterol and Low-Density Lipoprotein Cholesterol in the General Population. J Atheroscler Thromb.

[49] Whitworth JA, World Health Organization ISOHWG (2003). 2003 World Health Organization (WHO)/International Society of Hypertension (ISH) statement on management of hypertension. J Hypertens.

[50] Committee of the Japan Diabetes Society on the Diagnostic Criteria of Diabetes, M. *et al*. Report of the committee on the classification and diagnostic criteria of diabetes mellitus. *J Diabetes Investig* **1**, 212–228, 10.1111/j.2040-1124.2010.00074.x (2010). 10.1111/j.2040-1124.2010.00074.x PMC4020724 24843435

[51] Teramoto T (2007). Diagnostic criteria for dyslipidemia. Executive summary of Japan Atherosclerosis Society (JAS) guideline for diagnosis and prevention of atherosclerotic cardiovascular diseases for Japanese. J Atheroscler Thromb.

[52] Mitchell P, Smith W, Attebo K, Wang JJ (1995). Prevalence of age-related maculopathy in Australia. The Blue Mountains Eye Study. Ophthalmology.

[53] Klein R (1991). The Wisconsin age-related maculopathy grading system. Ophthalmology.

